# Comparison of Germ Cell Gene Expressions in Spontaneous Monolayer versus Embryoid Body Differentiation of Mouse Embryonic Stem Cells toward Germ Cells 

**DOI:** 10.22074/ijfs.2019.5557

**Published:** 2019-04-27

**Authors:** Maryam Gholamitabar Tabari, Seyed Gholam Ali Jorsaraei, Mohammad Ghasemzadeh-Hasankolaei, Ali Asghar Ahmadi, Masoumeh Ghasemi

**Affiliations:** 1 Infertility and Reproductive Health Research Center, Health Research Institute, Babol University of Medical Sciences, Babol, Iran; 2 Health Reproductive Research Center, Sari Branch, Islamic Azad University, Sari, Iran; 3 Cellular and Molecular Biology Research Center, Health Research Institute, Babol University of Medical Sciences, Babol, Iran

**Keywords:** Differentiation, Embryoid Body, Embryonic Stem Cells, Monolayer

## Abstract

**Background:**

Genetic and morphologic similarities between mouse embryonic stem cells (ESCs) and primordial germ cells (PGCs) make it difficult to distinguish differentiation of these two cell types *in vitro*. Using specific GC markers expressed in low level or even not expressed in ESCs- can help recognize differentiated cells *in vitro*. We attempted to differentiate the mouse ESCs into Gc-like cells spontaneously in monolayer and EB culture method.

**Materials and Methods:**

In this experimental study, we attempted to differentiate ESCs, Oct4-GFP OG2, into GC-like cells (GCLCs) spontaneously in two different ways, including: i. Spontaneous differentiation of ESCs in monolayer culture as (SP) and ii. Spontaneous differentiation of ESCs using embryoid body (EB) culture method as (EB+SP). During culture, expression level of four GC specific genes (*Fkbp6, Mov10l1, Riken* and *Tex13*) and *Mvh, Scp3, Stra8, Oct4* were evaluated.

**Results:**

In both groups, *Mov10l1* was down-regulated (P=0.3), while *Tex13* and *Riken* were up-regulated (P=0.3 and P=0.04, respectively). *Fkbp6* and *Stra8* were decreased in EB+SP and they were increased in SP group, while no significant difference was determined between them (P=0.1, P=0.07). Additionally, in SP group, gene expression of *Mvh* and *Scp3* were up-regulated and they had significant differences compared to EB+SP group (P=0.00 and P=0.01, respectively). *Oct4* was down-regulated in the both groups. Flow-cytometry analysis showed that mean number of Mvh-positive cells in the SP group was significantly greater compared to ESCs, EB+SP and EB7 groups (P=0.00, P=0.01, and P=0.3, respectively).

**Conclusion:**

These findings showed that ESCs were differentiated into GCLCs in both group. But spontaneous dif- ferentiation of ESCs into GCLCs in SP group (monolayer culture) compared to EB+SP (EB culture methods) has more ability to express GCs markers.

## Introduction

Embryonic stem cells (ESCs) can proliferate unlimitedly *in vitro* and they are unique in their ability by growing as immortal cells, expressing high telomerase (1) and preserving a normal karyotype during multiple passages (2). The medium supplemented by myeloid leukemia inhibitory factor (LIF) causes the ESCs to remain in an undifferentiating state (3). Spontaneous differentiation of ESCs can be easily triggered by the withdrawal of LIF from the medium culture of embryoid body (EB) *in vitro* (4, 5) and monolayer cells (6). EBs can be developed by aggregation of ESCs in suspension or hanging drops. They are rounded and three-dimensional structures which can generate populations of cells expressing genes indicative of lineages from all three germ layers (7). Under certain conditions, ESCs can also differentiate into different cell types such as neural progenitors (8), primordial germ cells (PGCs) (9), pancreatic linage (5) and blood cells (6). Over the past several decades, researchers have attained significant results in designing an appropriate *in vitro* model for the differentiation of ESCs into GCs (10, 11). It seems that these ESC-derived PGCs have the ability to enter meiosis as male and female gametes. However, compared to endogenous GCs, they do not undergo normal meiosis or become a functional gamete (12). Defects in natural and complete meiosis are one of the obstacles in achieving functional gametes.

In mice, over 53 genes are involved in the regulation of cell cycle (13). In a spontaneous differentiation protocol, expression of the GC markers was demonstrated (14). With regard to the literature, it can be suggested that continuing ESC culture in monolayer system for more than 10 days would lead to an increase in the GC marker expressions (15). Induced pluripotent stem cells express male GC genes during their spontaneous differentiation through EB formation (16).

Genetic and morphologic similarities between ESCs and PGCs make it difficult to diagnose these two cell type differentiations *in vitro*. Some specific GC markers, such as *4930432K21Rik, Mov10l1, Fkbp6* and *Tex13*, are expressed in reproductive system. Despite the low expression level of these genes in ESCs, they are highly expressed in PGCs (17). This will facilitate tracing differentiated cells *in vitro*. The Moloney leukemia virus 10-like 1 (*Mov10l1*) is a GC-specific autosomal gene in the mouse spermatogonia cells (18). *4930432K21Rik* is a new gene expressed in PGCs and gametes (17). *Fkbp6* is expressed in mouse testis (19). In human, mutations of this gene have been associated with male infertility (20). In mouse, Tex13 is also an X-linked gene, expressed in a GC-specific manner beginning at the spermatogonia stage (21, 22). In the present study, we attempted to differentiate the mouse ESCs, Oct4-GFP, into GC-like cells (GCLCs) spontaneously in two different ways: i. Spontaneous differentiation of ESCs in monolayer culture (SP) group and ii. Spontaneous differentiation of ESCs in EB culture method as (EB+SP) group. We tried to evaluate and compare expression level of GC specific genes in both groups, during culture *in vitro*.

## Materials and Methods

### Animals

Eight healthy adult NMRI mice, weighting more than 30 g, were usually housed in a light cycle of 12 hours light (6:00 AM to 6:00 PM) and 12 hours dark. Mice were obtained from the Animal Research Unit, Babol Medical University, Babol. Animal care and handling was done based on Animal Research Unit following the approval of Ethics Committee (Babol Medical University, Iran; MUBABOL.REC.1393.7).

### Study design

In this experimental study, samples were classified to two groups: i. Spontaneous differentiation of ESCs without LIF in its feeder cells (MEF) for 14 days as a monolayer culture (SP) group and ii. Spontaneous differentiation of ESCs using EB method. After 3 days culture for hanging drop and 4 days in bacterial plate, totally 7 days known as (EB7), single EB cells were cultured for 7 more days without LIF, totally 14 days; the latter group was named as EB culture methods (EB+SP).

### Cell culture

The utilized medium for MEF culture was a knock out- Dulbecco’s modified Eagle’s medium (DMEM, Gibco, UK) containing 15% fetal bovine serum (FBS, Bio west, USA), Pen/Str/Glu 100 U/ml , 100 mM non-essential amino acids and 0.1 mM 2-Mercaptoethanol (all from Gibco, UK). The applied medium for ESCs culture contain knock out-DMEM, 15% knock out-SR (Gibco, UK), Pen/Str/Glu 100 U/ml, 100 mM non-essential amino acids, 0.1 mM 2-Mercaptoethanol and 1000 IU/ml LIF (Chemicon, UK). ESCs differentiation and EB medium was similar to ESC medium, without LIF.

### Culture of mouse embryonic fibroblast

ESCs need feeder layer for growth. We cultured E13.5 mice in our study (23). Briefly, two female and one male mouse were put together in the same cage to mate. The morning after mating, vaginal plugs were checked and pregnant mice were identified (24) and scored as E0.5. After 13 days, pregnant mice were sacrificed to extract embryos. Embryos were isolated and washed, and then head and liver were separated from embryo and crushed using an 18-gauge needle, followed by culturing in MEF media. After two passages, MEF cells were ready for inactivation using incubation with 10 μg/ml mitomycin C (Sigma, Germany) for 1.5-2 hours. We used the cultured cells with passages 2-4, in this work.

### Culture and passage of mouse embryonic stem cells

The OG2 (ΔPE-GFP) ESC line (a kind gift from Dr. Sabour, Max Planck institute, Germany) was used in this study. Briefly, mouse ESC line was cultured on mitomycin C-treated MEFs with 0.1% gelatin-coated 25-cm^2^ flasks in ESC medium. Undifferentiated ESCs were cultured at 37˚C, 5% CO2 and 95% humidity. The medium was renewed daily. Seven two hours after primary culture, when the colony size was increased, cultured cells were trypsinized and expanded at a ratio of 1:3 on fresh feeder cells. The medium was changed every day.

### Embryonic stem cells differentiation *in vitro*

ESC colonies were dissociated with 0.25% trypsin-EDTA (Invitrogen, USA) (23). In order to remove MEFs from ESCs, we used the MEF faster reattachment potential, compared to ESC. After two rounds of reseeding (about 30-40 minutes), EB formation was induced with hanging drop prepared with a cell suspension containing 150-200 ESCs per 25 μl of mouse EB differentiation media for 3 days. EBs were next collected and transferred into non-attachment 10-cm^2^ bacterial dish for 4 additional days. EBs on seventh day were dissociated and digested by treatment with collagenase IV (0.01%), in order to obtain cell suspensions. The cells were then filtered and seeded in a gelatinized dish at about 20,000 cells per cm^2^. EB+SP groups were fed daily with EB medium for additional 7 days. On the other hand, single ESCs were seeded in a gelatinized dish at about 20,000 cells per cm^2^, for SP group. The cells were fed daily with ESC cell medium without LIF, for 14 days.

### Morphological evaluation

Morphological changes of differentiated cells were assessed by invert microscope (Nikon, Japan) during and after 14 days differentiation *in vitro*.

### RNA isolation and quantitative reverse-transcription polymerase chain reaction

Total RNA was isolated from ESCs, MEF cells, spontaneous differentiation of GCLCs (SP), 7 day EBs (EB7), spontaneous differentiation after EB formation (EB+SP) of GCLCs and somatic tissues of the testis and brain using RNA Isolation Kit (Roche, Germany). DNase I was used to eliminate genomic DNA contamination. RNA quality was determined using a Nano drop 2000c (Thermo Scientific, USA). cDNA was prepared in a total volume of 10 μl using a cDNA synthesis kit (TaKaRa, Japan) according to the manufacturer’s protocol. Target gene expressions were normalized based on the mouse housekeeping gene, Hprt. Gene transcripts were determined using SYBR Green I PCR Master Mix (Applied Biosystems, USA) containing 150 nmol of each forward (F) and reverse (R) primers (Table 1). Quantitative reverse-transcription polymerase chain reaction (qRT-PCR) analysis was performed using the ABI 7300 (Applied Biosystems). Relative quantification of gene expression was calculated using 2-ΔΔCt method. Three technical replicates were used for each qRT-PCR reaction. No template control (blank test) was served as a negative control. In all reactions, mouse testis and brain were respectively used as positive and negative controls.

**Table 1:** Primer sequences for quantitative reverse-transcription polymerase chain reaction


Gene	Primer sequence (5´→3´)	Length (bp)

Oct4	F: TGTTCCCGTCACTGCTCTGG	82
	R: TTGCCTTGGCTCACAGCATC	
Sycp3	F: GCAGTCTAGAATTGTTCAGAGCCAGA	75
	R: TCCAAACTCTTTATGAACTGCTCGTG	
Vasa	F: GGAGAGAGAGCAAGCTCTTGGAGA	74
	R: TGGCAGCCACTGAAGTAGCAA	
Stra8	F: GACGTGGCAAGTTTCCTGGAC	81
	R: TTCTGAGTTGCAGGTGGCAAA	
Tex13	F: GCCACAGGAAGACCGAATGAG	156
	R: TCTCTGCCTTTTCAGGGGATA	
Fkbp6	F: CCCCTCATCCCGCCAAATG	163
	R: TGCCAAACTCCCTCTCAGTTG	
Mov10l1	F: CGCTGTGACGAGTACAGTG	155
	R: CTGACAACCCTTTGCTAGAGTTT	
4930432K21Rik	F: AGAGAGTCGGAAGACAGCTCA	144
	R: CAGGGGGACCAGCTCTTTG	
Hprt	F: GTTAAGCAGTACAGCCCCAAA	140
	R: AGGGCATATCCAACAACAAACTT	


### Immunofluorescent staining

ESCs were cultured in two wells chamber slides at the end of previous culture step. They were fixed in 100% methanol (chilled at-20°C) at room temperature for 5 minutes. The cells were then heated in antigen retrieval buffer (100 mM Tris, 5% (w/v) urea, pH=9.5) at 95°C for 10 minutes, in order to obtaining optimal performance of certain antibodies. The cells were incubated for 10 minutes in phosphate buffer saline (PBS, Merk, USA) containing 0.1-0.25% TritonX-100 (ICN) for permeabilization. Subsequently, the cells were incubated with 1% bovine serum albumin (BSA, Bio west, USA), 22.52 mg/ml glycine in PBST (PBS+0.1% Tween 20) for 30 minutes to block non-specific binding of the antibodies. The cells were then overnight incubated with diluted Mvh primary antibody (1/100, Abcam13840, UK) in 1% BSA in PBST, using a humidified chamber at 4°. The cells were incubated with the secondary antibody (goat anti-rabbit IgG-PE: sc-3739,1/100, Santacruze, USA) in 1% BSA for 1 hour at room temperature in dark place, followed by incubation with 0.1-1 μg/ml DAPI (DNA stain, Sigma, USA) for 1 minute. Cover slips were mounted with a drop of mounting medium. Finally, the cells were evaluated under an inverted fluorescence microscope (Canada smart, Canada).

### Immunohistological examination

After specimen preparation of testis on the slides, they were preserved at room temperature. Slides were washed three times in TPBS (tween PBS) for 5 minutes. It was each time followed by immersing Triton X-100 (0.2% for cytoplasmic antigen) for 20 minutes. Blocking was performed using 10% normal serum with 1% BSA in PBST for 2 hours at room temperature, followed by adding 1% BSA and Mvh primary antibody (1/100) diluted in PBST and overnight incubation at 4°C in the dark. Fluorochrome-labeled secondary antibody (goat anti-rabbit IgG-PE: sc-3739;1/100, Santacruze, USA), diluted in TPBS containing 1% BSA, was applied to the slide and incubated for 1 hour at room temperature in dark. The coverslip was mounted using a compatible mounting medium.

### Flow-cytometry analysis

Following differentiation, the cells were fixed before intracellular staining. Fixation was occurred by placing the cells in 0.01% formaldehyde for 10-15 minutes. One hundred microliter of detergent-based permeabilizing agent Triton x100 (0.1-1% in PBS) was added and the cells were incubated in dark at room temperature for 15 minutes. Mvh primary antibody (0.1-10 μg/ml) was added and the cells were incubated for at least 30 minutes at 4°C in dark. The fluorochrome-labeled secondary antibody was diluted in 3% BSA/PBS at the optimal dilution (1:100-1:400) and added to the cells. This was followed by incubation for at least 20-30 minutes at 4°C in the dark. The cells were suspended in ice cold PBS, 3% BSA and 1% sodium azide. Secondary antibody IgG-PE was detected using the FL1 channel of the FACS Calibur TM flow-cytometer (BD Biosciences, USA) and the percentage of positive cells was measured by the FlowJo software.

### Statistics

All experiments were independently repeated at least three times. Data are presented as mean ± SD. Statistical analysis was determined using ANOVA, independent t test. All statistical tests were performed using SPSS (Statistical Package for the Social Sciences, version 20, SPSS Inc., USA) software. Flow cytometry data were analyzed using FlowJo 7.6 software. A P<0.05 was considered significant.

## Results

### Morphological evaluation

**Fig 1:**
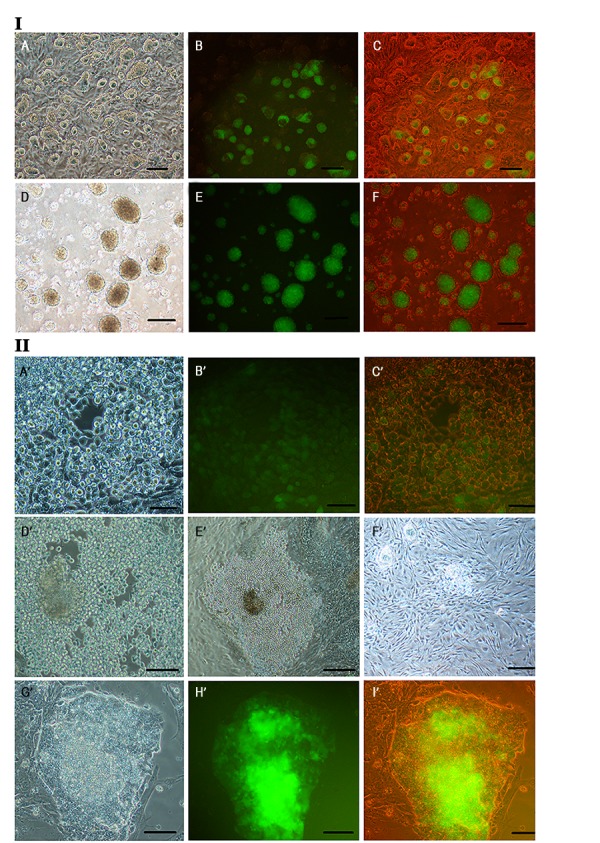
Morphological assessment of Oct4-GFP embryonic stems cell (ESC) colonies, at day 7th of embryoid body (EB) culture (EB 7). **I: A.** Bright-field image of Oct4-GFP ESC colonies growing on an embryonic fibroblast feeder layer, **B.** Fluorescent image of Oct4-GFP ESC colonies show Oct4 expression with green color, **C.** Merged fluorescent and bright-field images of Oct4-GFP ESCs, **D.** Bright-field image of EB colonies after 7 days suspension culture in bacterial plate, **E.** Fluorescent image of EB aggregates show Oct4 expression in green, and **F.** Merged fluorescent and bright-field images of EB aggregates (A-C: ×10), (D-F: ×4) (scale bar: 100 μm). **II. A'.** Bright-field image of germ cell like cells (GCLCs) after 7 days, without feeder cells in (EB+SP) group, **B'.** Fluorescent image of Oct4-GFP GCLCs show Oct4 expression with green color, **C'.** Merged fluorescent and bright-field image, **D', E'.** Bright-field image of differentiated cells after culture of EB aggregates for 7 days, without feeder cells in ESC differentiation medium, **F'.** Bright-field image of ESCs colony during differentiation in SP group, **G'.** Bright-field image of differentiated cells after 14 days culture of singled ESCs in SP group, **H'.** Fluorescent image of section G shows Oct4 expression in green, and **I'.** Merged fluorescent and bright-field images of section G (Aˊ-Cˊ, Fˊ-Hˊ, Iˊ: ×20), (Dˊ: ×10 and Eˊ: ×4) (scale bar: Aˊ-Dˊ, Fˊ: 10 μm, Eˊ, Gˊ-Iˊ: 100 μm).

Figure 1I.A-C shows ESC colonies after 48 hours culture *in vitro*. Colonies were dense with distinct and tight borders, while individual cells were not visible. Colonies did not touch each other. Figure 1.I.D-F shows EB aggregation with consistently round-shapes. Morphological evaluation of these cells during culture in EB+SP group shows that those small and single EB cells were changed into round-shapes.

Single cells in tissue culture plate attached and formed an integrated and fabric building tightly stuck to the bottom of dish. This made trypsinization process very hard (Fig .1II.Aˊ-Cˊ). We also cultured and differentiated EB aggregation on day seven, without dissociating to the single cell (aggregated EB), in tissue culture plate just to look up and compare. After culture of aggregated EB for 7 days in ESC differentiation medium, we saw loose cell-cell adhesions in the colonies and the cells shape was changed to round-shape as well as single EB cells (Fig .1II.Dˊ, Eˊ). In SP group, ESCs colonies were also changed to form round-shaped cells. They were slowly separated from the colony, while clinging tightly to their feeder layer (Fig .1II.Fˊ). Differentiated mESCs on MEFs showed that colonies were merged and lost border integrity (Fig .1II.Gˊ-Iˊ).

### Expression of germ cell-specific genes

**Fig 2:**
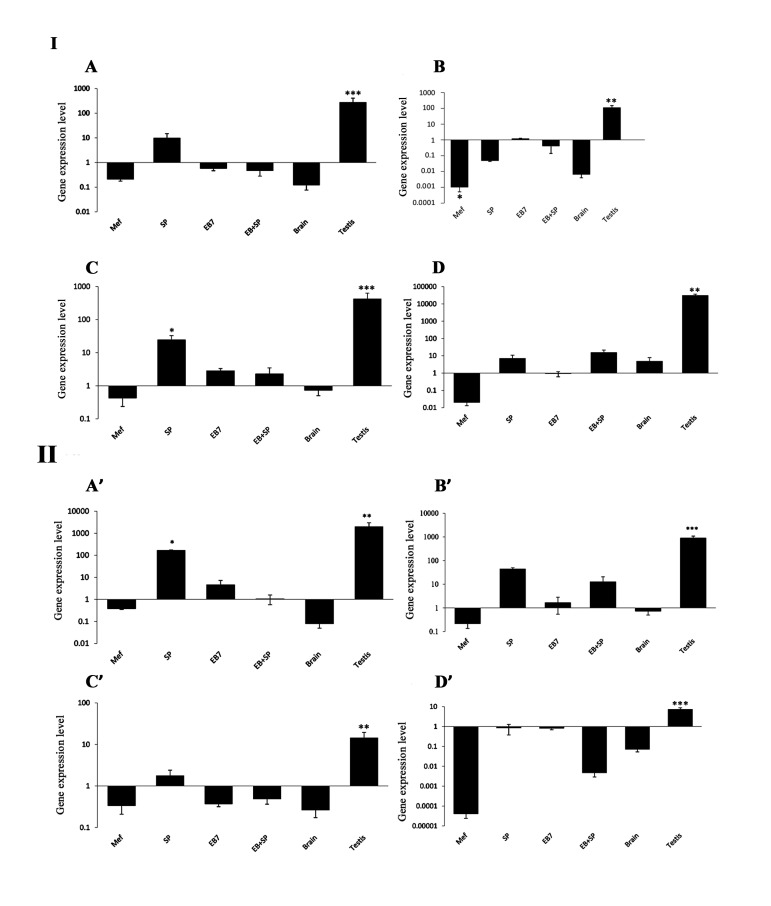
Quantitative reverse-transcription polymerase chain reaction (qRT-PCR) in embryonic stems cell (ESC)-derived cells of study groups. **I:** Gene expression level of specific germ cell markers (**A.** *Fkbp6,* **B.** *Mov10l1,* **C.** *4930432K21Rik,* and **D.** *Tex13*) in ESC-derived cells of mouse embryonic fibroblast (MEF), SP, day 7 of embryoid body (EB) culture (EB7), spontaneous differentiation after EB formation (EB+SP), brain as negative control and testis as positive control compared to ESCs. **II:** Gene expression level of **A'.** *Mvh,* **B'.** *Scp3,* **C'.** *Stra8*, and **D'.** *Oct4* in ESC-derived cells of MEF, SP, day 7 of EB culture (EB7), spontaneous differentiation after EB formation (EB+SP), brain as negative control and testis as positive control compared to ESCs. Values are mean ± SD. *; P<0.05, **; P<0.01, ***; P<0.001. The amount of the undifferentiated mESC is normalized to 1.

The expression levels of four GC-related genes (*Fkbp6, Mov10l1, 4930432K21Rik* and *Tex13*) as well as *Oct4, Mvh, scp3, stra8* and *HPRT* was determined by qRT-PCR. These findings were confirmed by determining their expression in mouse brain (as a negative control) and testis (as a positive control) somatic tissues. The expression levels of above GC markers were compared in the two study groups: i. SP and ii. EB+SP. Gene expression levels between different groups indicated some variations. qRT-PCR showed that in the both groups, expression of *Mov10l1* was down-regulated and there was no significant difference between them (P=0.3). Tex13 was up-regulated in both groups, but there was no significant difference between them (P=0.3). Riken was up-regulated in both groups and this elevation was significantly higher in SP group compared to EB+SP (P=0.04). *Fkbp6* was down-regulated in EB+SP and up-regulated in SP groups with no significant difference between them (P=0.1, Fig .2).

**Fig 3:**
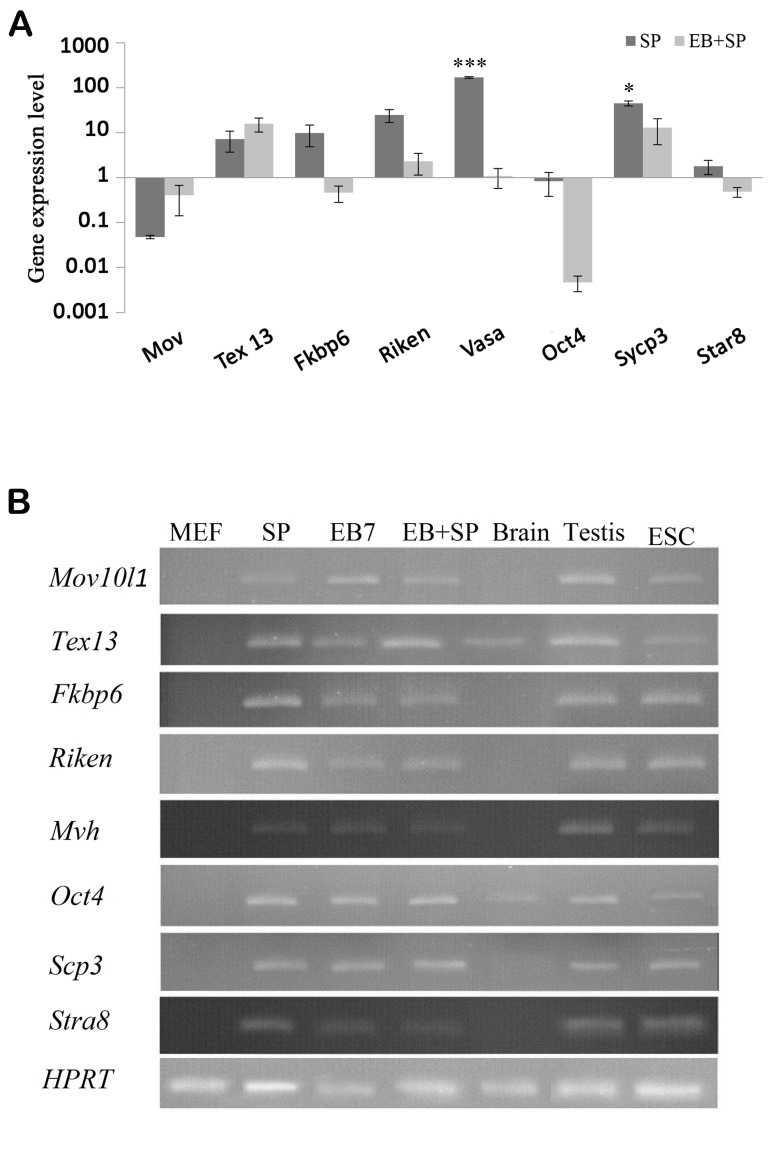
Comparison of meiotic marker gene expression levels. **A.** Graph shows expression level of *Fkbp6, Mov10l1, 4930432K21Rik, Tex13, Mvh, Scp3,* and *Stra8* in SP and embryoid body (EB) EB+SP groups. The amount of the undifferentiated mouse embryonic stems cell (ESC) mESC is normalized to1 and **B.** Graph shows expression of germ-cell genes during ESCs differentiation. RNA was isolated from mouse embryonic fibroblast (MEF), SP, EB7, EB+SP cells, brain and adult testis tissues as well as ESCs, for Quantitative reverse-transcription polymerase chain reaction (qRT-PCR). Values are mean ± SD. *; P<0.05 and ***; P<0.001.

*Vasa* and *Scp3* were up-regulated in both groups, while it was increased with significant difference in SP group, compared to EB+SP (P=0.00 and P=0.01, respectively). Additionally *Oct4* in both groups and *Stra8* in EB+SP group were decreased, while no significant difference was observed between them (P=0.1 and P=0.1, respectively). *Oct4* level was down-regulated in all study groups, compared to ESCs (P<0.05, Fig .3A).

Four GC-specific genes (*Fkbp6, Mov10l1, 4930432K21Rik,* and *Tex13*) were analyzed in differentiated cells. All GC-specific genes (except Mov10l1) were expressed at moderate levels in SP group and they had no or low expression level in EB+SP groups. *Fkbp6, Mov10l1, Tex13* and *4930432K21Rik* were expressed at moderate-to-high levels in adult testis. In addition, *Fkbp6, Mov10l1, 4930432K21Rik* and *Tex13* exhibited very low or no expression in brain tissues (Fig .3B).

It was found that *4930432K21Rik* was expressed in higher level than other genes in SP group. It was approximately 617-fold higher than that of *Mov10L1*, 3.4- fold higher than that of *Tex13* and 2.4-fold higher than that of *Fkbp6* in SP group. However, in EB+SP group, *Tex13* was expressed in higher level than other genes. It was approximately 39.2-fold higher than that of *Mov10L1,* 6.8- fold higher than that of *4930432K21Rik,* and 39.2-fold higher than that of *Fkbp6*.

### Immunostaning

**Fig 4:**
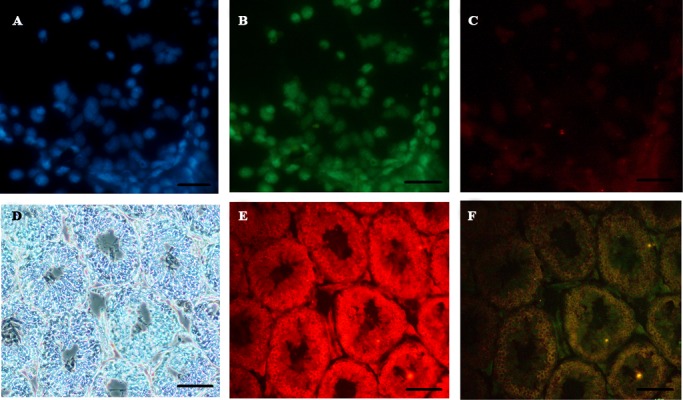
Cytoplasmic protein expression analysis of Mvh in embryonic stems cell (ESC)-derived differentiated cells and mouse adult testis, using immunofluorescent staining. **A.** Image shows that nuclei were stained with DAPI (in blue), **B.** Oct4-GFP expression (in green), **C.** Anti-Mvh antibody as a germ cell (GC) marker (in red) (scale bar: 10 μm), **D.** Image shows section of mouse adult testis in bright-field, E. Anti-Mvh antibody as a GC marker (in red), and F. When the light of the microscope is off and the fluorescent light is on (in dark) (scale bar: 100 μm).

To show the expression of Mvh (Vasa, Ddx4) protein, as a GC marker, in differentiated cells and testis tissue (positive control), primary and secondary antibodies staining was performed. Fluorescent microscope analysis showed positive red color due to the expression of Mvh protein. The nucleus of defined GCLC round cells were counterstained with DAPI. We observed these round-shaped cells were red, indicating expression of Vasa protein. This coloration in the cells of SP group was greater than that of EB+SP group (Fig .4).

### Flowcytometry

**Fig 5:**
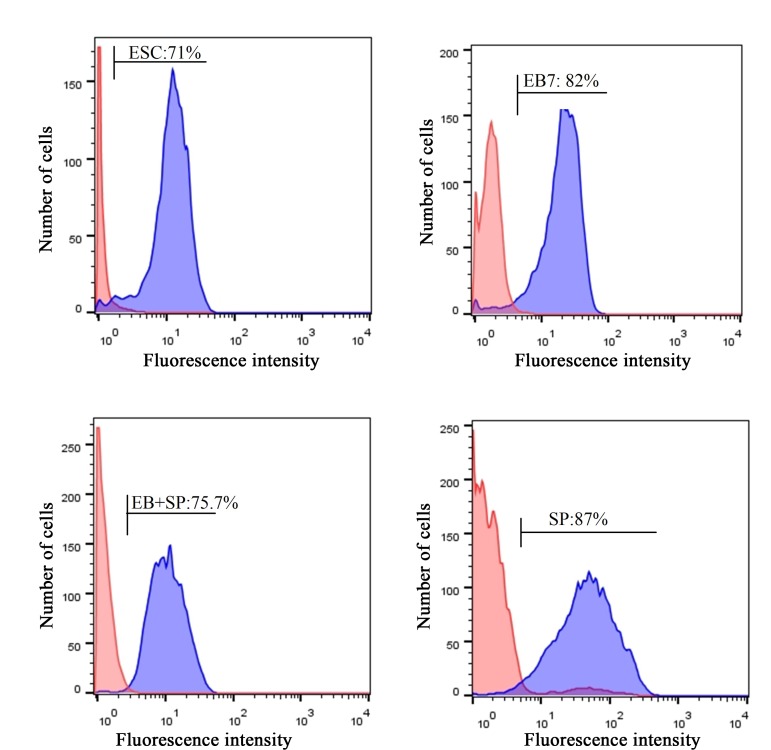
Flow-cytometer analysis for the expression level of Mvh in study groups. Expression level in undifferentiated embryonic stems cell (ESC), spontaneous differentiation of ESCs in monolayer culture (SP), day 7 of embryoid body (EB) culture (EB7), spontaneous differentiation of ESCs in EB culture method (EB+SP).

Since qRT-PCR showed that the expression of GC markers were enhanced in differentiated cells, we investigated the protein expression of Mvh by flow-cytometer. Mean fluorescent intensity (MFI) of the cells showed significantly higher Mvh positive cells in the SP group (87.2 ± 2.61) compared to undifferentiated ESC (71 ± 3.02) and EB+SP (75.74 ± 3.90) groups (P=0.00 and P=0.01, respectively). However, compared to EB7 group (82 ± 2.61), Mvh increased MFI of the cells was not significantly different (P=0.3, Fig .5).

## Discussion

In this study, morphological evaluation showed that stem cell colony formed round-shaped cells, tightly sticking to the bottom of dish, as with Nagai et al. (25) report indicating that PGCs have round-oval shapes with large size and large nucleus. In this study, GC specific genes (*Fkbp6, Mov10l1, 4930432K21Rik* and *Tex13*) displayed different levels of gene expression in GCLCs (both groups), somatic tissues and ESCs. Expression of specific GC markers, with no or low level in ESCs, can appropriately facilitate recognition of differentiated cells *in vitro*. This study showed that expression of the GC genes in SP group (monolayer) were more than EB+SP (EB system). In terms of gene expression patterns, monolayer culture condition was suggested to be superior to the EB culture system (26). Monolayer culture system was also suggested as a more appropriate protocol to promote female GC (23) and neural differentiations (8). On the other hand, Talaei-Khozani et al. (27) showed that in spontaneous differentiation, when no growth factors were used, the expression of meiosis markers in EB method were greater than monolayer culture system. The expression of *Mov10l1* gene was decreased in SP and EB+SP groups. There was no significant difference between expression levels of these two groups. Expression of this gene was increased in the leptotene and zygotene stages of meiosis and decreased at the end of meiosis (28). After 21 postnatal days (dpp), this gene transcription frequency was continuously reduced, coinciding with the emergence of the first generation of rounded spermatozoa (29).

Results of this study showed that *Tex13* was expressed in GCLCs of SP and EB+SP groups. This gene was expressed in the embryo of 12.5 dpp male mice, as well as the testicular tissue and sperm cells (17). Tex13 is an X-link gene expressed in the early stages of spermatocyte, during the leptotene and zygotene stages of meiosis (28). However, it appears to undergo translational suppression before late meiosis. Our results showed that *Fkbp6* was expressed in GCLCs of SP, but not EB+SP group. Mouse *Fkbp6* is not involved in the initiation of synapsis. It plays role in monitoring progression and/or maintaining synapsis between homologous pairs (30). However, deficient Fkbp6 male mice were completely sterile and they had abnormal pachytene spermatocytes which failed to proceed beyond the pachytene stage (31).

In this study, *4930432K21Rik* expression level was elevated in both groups, while the level of expression was not significantly different compared to EB+SP group. It is worthy to note that expression of this gene was higher than the other three above genes. *4930432K21Rik* is a new gene with unknown function expressed in PGCs and gametes of mouse embryos (17). Expression level of *Mvh,* as a sex-linked gene, in SP cells was significantly increased in comparison with EB+SP cells. The expression of this gene was increased with the onset of meiosis and remained high up to the end of spermatogenesis (28). Absence of *Mvh* results in the arrest of zygotene stage in male gametes (32). Analysis of Mvh expression at the protein level, using flow-cytometer, showed a greater increase in the SP group rather than other groups, confirming the findings obtained from RT-PCR. Mvh is a cytoplasmic protein and product of the Vasa homolog gene, induced by somatic cell of the genital crest, and it remains until formation of the post meiotic GCs. Hence, mutation in this gene leads to defect in proliferation and differentiation of PGCs (33).

The current study demonstrated down-regulation of *Oct4* in all of the studied groups, compared to ESCs. *Oct4* is a well-known factor, which plays major role in pluripotency maintenance of ESCs. *Oct4* begin to show high expression level in the inner cell mass, but its level is decreased as the cells enter to epiblast stag. Oct4 expression level of GCs in vivo is high until E13.5. Then, it is decreased in the zygote/pachytene stages of first meiosis around E16.5 (34). In this study, expression level of Sycp3 was obviously higher in the SP, rather than other groups. This gene is essential for synaptoneal complexes, chromosomal synapse and male fertility (35). The chromosomes lacking this gene are not able to form synaptic complexes (34). This event caused Sycp3-/- mice to stop development at zygote stage (36). Our findings showed that expression of *Stra8* gene was slightly increased in the SP group and decreased in the EB+SP group. Male and female mice that do not express Stra8 are infertile (37). Expression of this gene is initially determined in immature testis as well as GCs with mitotic activity after birth, and then its expression is increased in undifferentiated GCs of adult testis (38).

Our result showed that after terminating culture, mESCs were differentiated and changed into the round-shape cells with relatively large and distinct nuclei. In this study, some GC gene expression and meiosis marker were increased in both groups. The level of GCs-specific gene expressions in SP group was higher than EB+SP group. *Oct4* and *Mov10l1* were down-regulated and *Tex13, Riken, Fkbp6, Vasa, Stra8* and *Scp3* were up-regulated in the SP group after differentiation. Furthermore, expression levels of Mov10l1, *Fkbp6, Oct4* and *Stra8* were down-regulated in EB+SP group, while *Tex13, Riken, Vasa* and *Sycp3* were up-regulated. On the other hand, findings obtained from flow-cytometry indicated that SP group cells had more MFI in Vasa protein than EB+SP group cells.

## Conclusion

Oct4 down-regulation, as a pluripotency factor, and expression of meiosis markers indicated that ESCs were successfully differentiated into GCLCs in both groups. Evaluation of gene expression patterns in both groups demonstrated that monolayer culture was more efficient to produce GCLCs, in compression with EB methods. Further recruitment of culture conditions and optimization will still be needed for successful and high quality GCs differentiation *in vitro*.
